# Prediction of atelectasis in *Mycoplasma pneumoniae* pneumonia using a SHapley Additive exPlanations-interpretable machine learning model

**DOI:** 10.3389/fped.2026.1747948

**Published:** 2026-05-11

**Authors:** Jia Sun, Tengfei Wang, Mengsi Li, Mian Wang

**Affiliations:** 1 Department of Pediatrics, Wuhan Wuchang Hospital, Wuhan, China; 2 School of Transportation and Logistics Engineering, Wuhan University of Technology, Wuhan, China

**Keywords:** atelectasis, *Mycoplasma pneumoniae* pneumonia, neural network, risk prediction, SHAP

## Abstract

**Objective:**

This study aimed to evaluate the performance of three machine learning models—K-nearest neighbors (KNN), support vector machine (SVM), and neural network (NN)—for predicting the risk of atelectasis in children with *Mycoplasma pneumoniae* pneumonia (MPP). The study also incorporated SHAP (SHapley Additive exPlanations) analysis to interpret model predictions.

**Methods:**

Based on the clinical data of 508 pediatric patients, we performed feature selection and developed KNN, SVM, and NN models. Model performance was compared on an independent validation set, and SHAP values were employed to elucidate the predictive logic of the models.

**Results:**

On the validation set, the neural network (NN) model demonstrated the best overall performance, with an AUC of 0.89 and an accuracy of 0.82. The KNN model showed comparable performance (AUC = 0.88), while the SVM model achieved the highest specificity (0.87). The SHAP analysis consistently identified neutrophil percentage (NEU.pct), serum amyloid A (SAA), and C-reactive protein (CRP) as the most critical variables influencing the predictions.

**Conclusion:**

This study demonstrates the effectiveness of different machine learning models in predicting the risk of atelectasis in MPP. The neural network, in particular, exhibited superior performance owing to its powerful non-linear modeling capabilities. These interpretable models provide clinicians with a diverse set of tools to accommodate various clinical priorities, such as overall accuracy or high specificity, thereby facilitating the early identification and stratified management of high-risk children.

## Introduction

*Mycoplasma pneumoniae* (MP) is a primary cause of community-acquired pneumonia (CAP) in children, responsible for 10%–40% of hospitalizations, especially among school-aged children (1). While *Mycoplasma pneumoniae* pneumonia (MPP) is typically mild and self-limiting, a significant subset of 10%–30% of patients progress to severe or refractory disease. This severe form can lead to debilitating complications such as bronchial stenosis, bronchiectasis, pleural effusion, and atelectasis, which not only worsen clinical outcomes and extend hospital stays but also pose a risk of long-term pulmonary dysfunction and increased healthcare burden (2, 3). Therefore, the early identification of atelectasis in pediatric MPP is critical for timely intervention and improving prognoses.

Among these complications, atelectasis is particularly concerning due to its profound impact on respiratory function. It is characterized radiologically by patchy or segmental opacities and potential mediastinal shifts. Clinically, children with MPP complicated by atelectasis often present with persistent fever, markedly elevated inflammatory markers, and frequently require invasive interventions like fiberoptic bronchoscopy to facilitate recovery (4, 5). Although prior retrospective studies have underscored the importance of early identification (6–9), they have largely relied on traditional univariate or multivariate logistic regression, and no robust risk prediction model has been established to date.

Current diagnostic methods for atelectasis have significant limitations. While bronchoscopy is effective for airway clearance, mucus plug removal, and promoting lung re-expansion, it is an invasive procedure with inherent risks, and its early application remains controversial due to a lack of standardized criteria (5, 10, 11). Furthermore, its complexity and the scarcity of expertise in primary care settings restrict its widespread use. Alternatively, chest computed tomography (CT) offers superior diagnostic detail for identifying mucus plugs and airway obstruction sites (12, 13). However, concerns over radiation exposure and patient compliance limit the feasibility of repeated CT scans. This diagnostic dilemma highlights an urgent need for a non-invasive and reliable tool for the early prediction of atelectasis in children with MPP.

Recent advancements in computational technology and artificial intelligence offer a promising solution. Machine learning (ML), a powerful data-driven approach, is increasingly being leveraged for disease diagnosis, prognosis, and risk prediction (14–16). A growing body of evidence demonstrates the superiority of ML models in handling complex, high-dimensional clinical data. For instance, ML has achieved excellent predictive performance in chronic diseases (AUCs 0.74–1.0) and high accuracy in predicting outcomes for pneumonia-related conditions, with AUCs reaching 0.97 (17, 18). Similarly, ML models have successfully predicted ventilator-associated pneumonia (AUROC 0.854) and have been utilized for pneumonia severity assessment and distinguishing COVID-19 from other pneumonias (19–22). These findings collectively suggest that ML holds significant potential for predicting atelectasis risk in pediatric MPP, a research area that, to our knowledge, remains unexplored.

ML offers a diverse set of tools to address this clinical challenge, with different algorithms providing unique advantages suited to various clinical data and predictive scenarios (23, 24). For instance, K-nearest neighbors (KNN) makes predictions based on historical data from similar cases; support vector machines (SVM) excel at handling multivariate data and can effectively delineate between high- and low-risk groups; while neural networks (NN) are capable of learning complex, non-linear patterns, demonstrating powerful predictive capabilities.

In the context of predicting atelectasis risk in children with MPP, selecting the most appropriate model is crucial. This study will systematically compare the performance of the three aforementioned models to identify the optimal predictive tool, with a particular focus on their clinical utility. To this end, we will employ the SHAP (SHapley Additive exPlanations) interpretability framework to clearly reveal the key clinical factors driving each prediction.

Our ultimate goal is to develop a decision support tool that clinicians can understand and trust. This tool is designed to enable early and precise risk stratification for atelectasis in children with MPP, thereby assisting physicians in identifying the optimal window for intervention, proactively reducing the incidence of complications, and ultimately improving patient outcomes.

## Methods

### Ethics approval

This study was approved by the Ethics Committee of Wuchang Hospital, Wuhan. This retrospective study was conducted using data from 508 pediatric patients with MPP who underwent chest CT scans at the Department of Pediatrics, Wuchang Hospital, Wuhan, between July 2022 and June 2024. The requirement for informed consent was waived due to the retrospective nature of the study.

### Study population

This retrospective modeling study aimed to develop ML models to predict the risk of atelectasis in pediatric patients with MPP, based on real-world clinical data. Pediatric cases admitted between July 2022 and June 2024 were screened. MPP was diagnosed according to the Guidelines for the Diagnosis and Treatment of Mycoplasma Pneumonia in Children (2023 Edition). Diagnosis required clinical or radiological evidence of pneumonia plus one laboratory criterion: (1) a ≥4-fold increase in IgG titers; (2) IgM titer ≥1:160; or (3) positive PCR for Mycoplasma pneumoniae. MPP with atelectasis was defined as MPP accompanied by radiologically confirmed segmental or lobar atelectasis. Patients were excluded if they had: (1) symptom duration >14 days; (2) co-infections with bacteria, viruses, or Mycobacterium tuberculosis; (3) underlying conditions including bronchiectasis, immotile cilia syndrome, immunodeficiency, or hematologic diseases; (4) concomitant renal, hepatic, cardiovascular, or connective tissue diseases; or (5) missing critical clinical data. Missing laboratory values were imputed as described below. Ultimately, 508 eligible patients were enrolled, comprising 297 non-atelectasis cases (58.5%) and 211 atelectasis cases (41.5%). Although this was a retrospective study without *a priori* sample size calculation, the final sample size (*n* = 508) was deemed sufficient given the limited number of features selected in the final models (4–7 features), yielding a high events-per-variable ratio and stable performance estimates with narrow confidence intervals.

### Inclusion and exclusion criteria

The diagnosis of MPP was strictly confirmed according to the diagnostic criteria outlined in the 8th edition of Zhu Futang Practical Pediatrics. All included cases were required to have a definitive MPP diagnosis (positive or negative) and at least one laboratory result or demographic variable. Any missing data in other features had to meet the criteria for imputation.

Exclusion criteria included: disease duration >14 days at admission; co-infection with bacteria, viruses, or tuberculosis; comorbid bronchiectasis, immotile cilia syndrome, immunodeficiency, hematological diseases, or chronic lung disease; severe renal, hepatic, cardiovascular, or connective tissue disorders; >30% missing data without possibility of imputation; or unclear labeling of the target variable.

### Data collection

A total of 20 candidate predictors were recorded for each patient at the time of admission (baseline), including two demographic factors (sex, age) and 18 laboratory or clinical indicators such as CRP, hs-CRP, SAA, WBC, NEU, NEU%, LYM, PLT, ALT, AST, CK-MB, LDH, Ferritin, IL-6, PCT, IgE, fever duration, and maximum body temperature. The diagnosis of atelectasis was confirmed based on chest CT findings. Radiological criteria included direct signs such as lung volume loss, increased attenuation or opacity, and displacement of interlobar fissures, mediastinum, or hilum, as well as indirect signs such as crowded ribs. To minimize bias, all CT images were independently reviewed by two experienced radiologists who were blinded to the patients' clinical information and laboratory results. In cases where the initial assessments differed, a final diagnosis was reached through consensus discussion involving a third senior radiologist. The inter-observer reliability was excellent, with a Cohen's kappa coefficient of 0.72.

### Data preprocessing and statistical analysis

To ensure rigorous model evaluation and prevent data leakage, the dataset was first randomly stratified by outcome and divided into a training set (406 cases) and a validation set (102 cases) at an 8:2 ratio. Subsequently, data imputation was performed.

Variables with ≤30% missing values were imputed using multiple imputation via the random forest algorithm (miceforest package) (25). Subsequently, univariate analysis was conducted exclusively on the training set to identify significant features. Normality of continuous variables was assessed using the Kolmogorov–Smirnov test; normally distributed variables were compared using independent-sample t-tests, while non-normally distributed variables were compared using the Mann–Whitney U test. Categorical variables were analyzed using the chi-square or Fisher's exact test. Variables with a *P*-value <0.05 were considered statistically significant and retained for initial modeling.

For model development, ML models were trained on each of the five imputed training datasets. The reported performance metrics (e.g., AUC, accuracy) represent the averaged results of these five models evaluated on the corresponding validation sets.

### Feature selection and model development

To ensure model generalizability, all feature selection procedures were confined to the training set. Initially, models were trained using statistically significant variables, and feature importance was evaluated using SHAP. Subsequently, a forward stepwise selection algorithm was employed to iteratively incorporate variables. The inclusion of features was determined by their contribution to model performance, specifically measured by the AUC using five-fold cross-validation. This process continued until no further performance improvement was observed.

Three ML algorithms were evaluated: KNN, SVM, NN. Hyperparameters were tuned using Bayesian optimization, and nested cross-validation was applied to avoid overfitting.

As a non-parametric, instance-based method, KNN classifies a data point by majority vote of its K-nearest neighbors in the feature space. It requires no explicit training but is computationally intensive during inference and sensitive to the choice of k and feature scaling (26). SVM is a discriminative classifier that finds the optimal hyperplane to separate classes with the maximum margin. Its efficacy in non-linear classification is achieved through kernel functions, which implicitly map data into higher-dimensional spaces (27). In this study, we implemented a multi-layer perceptron (MLP) to capture complex, non-linear patterns for robust prediction. NN are composed of interconnected layers of neurons that learn hierarchical feature representations from data through a process of backpropagation. This architecture enables them to model highly complex, non-linear relationships, forming the basis for deep learning 1. The model hyperparameters were optimized using Bayesian optimization. The final architecture consisted of two hidden layers with 64 and 32 neurons, respectively. Both hidden and output layers utilized the sigmoid activation function. The model was trained via stochastic gradient descent (SGD) with a learning rate of 0.001. To mitigate overfitting, we applied L2 regularization with a penalty factor of 0.0001 combined with a dropout rate of 0.5. Training was conducted for a maximum of 200 epochs with a batch size of 32, employing an early stopping mechanism to halt the process when validation loss ceased to improve.

### Model evaluation

Model performance was evaluated on both the training and validation sets using multiple metrics. In addition to discrimination metrics—including accuracy, precision, recall, F1 score, area under the receiver operating characteristic curve (AUC), specificity, positive predictive value (PPV), and negative predictive value (NPV)—we also assessed calibration to evaluate the reliability of the predicted probabilities. Calibration was visualized using calibration curves and quantified using the Brier score (where lower scores indicate better calibration).

## Result

### Feature selection and model explainability

In the training set, univariate analysis identified 14 variables with statistically significant differences (*P* < 0.05) between patients with and without MPP-associated atelectasis, including fever duration (Fever.Dur), maximum temperature (Temp.Max), neutrophil count (NEU), lymphocyte count (LYM), neutrophil percentage (NEU.pct), serum amyloid A (SAA), C-reactive protein (CRP), high-sensitivity CRP (hs.CRP), platelet count (PLT), creatine kinase-MB (CK-MB), lactate dehydrogenase (LDH), ferritin, interleukin-6 (IL-6), and procalcitonin (PCT). Other variables such as sex, age, WBC, ALT, AST, and IgE did not reach statistical significance (Table 1).

**Table 1:** Baseline characteristics of participants.

Characteristics	Cohort 0 (*n* = 297)	Cohort 1 (*n* = 211)	p(U)
Sex			0.063
Female	133 (44.8%)	113 (53.6%)	
Male	164 (55.2%)	98 (46.4%)	
Age (years)	7.60 (5.50, 9.70)	8.00 (6.30, 9.50)	0.203
Fever.Dur (days)	4.00 (0.00, 6.00)	6.00 (4.00, 7.00)	<0.001
Temp.Max (°C)	38.50 (36.80, 39.00)	39.00 (38.70, 39.50)	<0.001
WBC (×10^9^/L)	6.83 (5.51, 8.12)	6.92 (5.67, 8.50)	0.258
NEU (×10^9^/L)	3.89 (3.00, 5.05)	4.41 (3.48, 5.75)	<0.001
LYM (×10^9^/L)	1.96 (1.58, 2.52)	1.70 (1.34, 2.10)	<0.001
NEU.pct (%)	0.60 (0.51, 0.66)	0.66 (0.58, 0.72)	<0.001
SAA (mg/L)	31.35 (10.43, 64.01)	99.44 (57.03, 160.14)	<0.001
CRP (mg/L)	4.45 (2.10, 7.61)	12.92 (8.43, 24.34)	<0.001
hs.CRP (mg/L)	6.45 (2.08, 10.75)	18.48 (11.32, 30.54)	<0.001
PLT (×10^9^/L)	250.00 (206.00, 295.00)	227.00 (189.50, 263.50)	<0.001
CK.MB (U/L)	3.30 (2.70, 4.10)	2.60 (1.90, 3.45)	<0.001
LDH (U/L)	247.50 (215.80, 272.10)	251.50 (223.45, 284.50)	0.012
ALT (U/L)	12.60 (10.20, 16.10)	12.40 (9.90, 16.60)	0.509
AST (U/L)	29.60 (24.70, 34.80)	30.00 (25.75, 34.55)	0.548
Ferr (μg/L)	90.31 (69.30, 118.12)	121.99 (93.25, 160.35)	<0.001
IL.6 (pg/mL)	12.86 (7.31, 20.22)	18.02 (11.04, 26.14)	<0.001
PCT (ng/mL)	0.06 (0.04, 0.09)	0.08 (0.05, 0.14)	<0.001
IgE (IU/mL)	81.00 (32.70, 239.88)	88.48 (35.00, 261.24)	0.324

**Figure 1:**
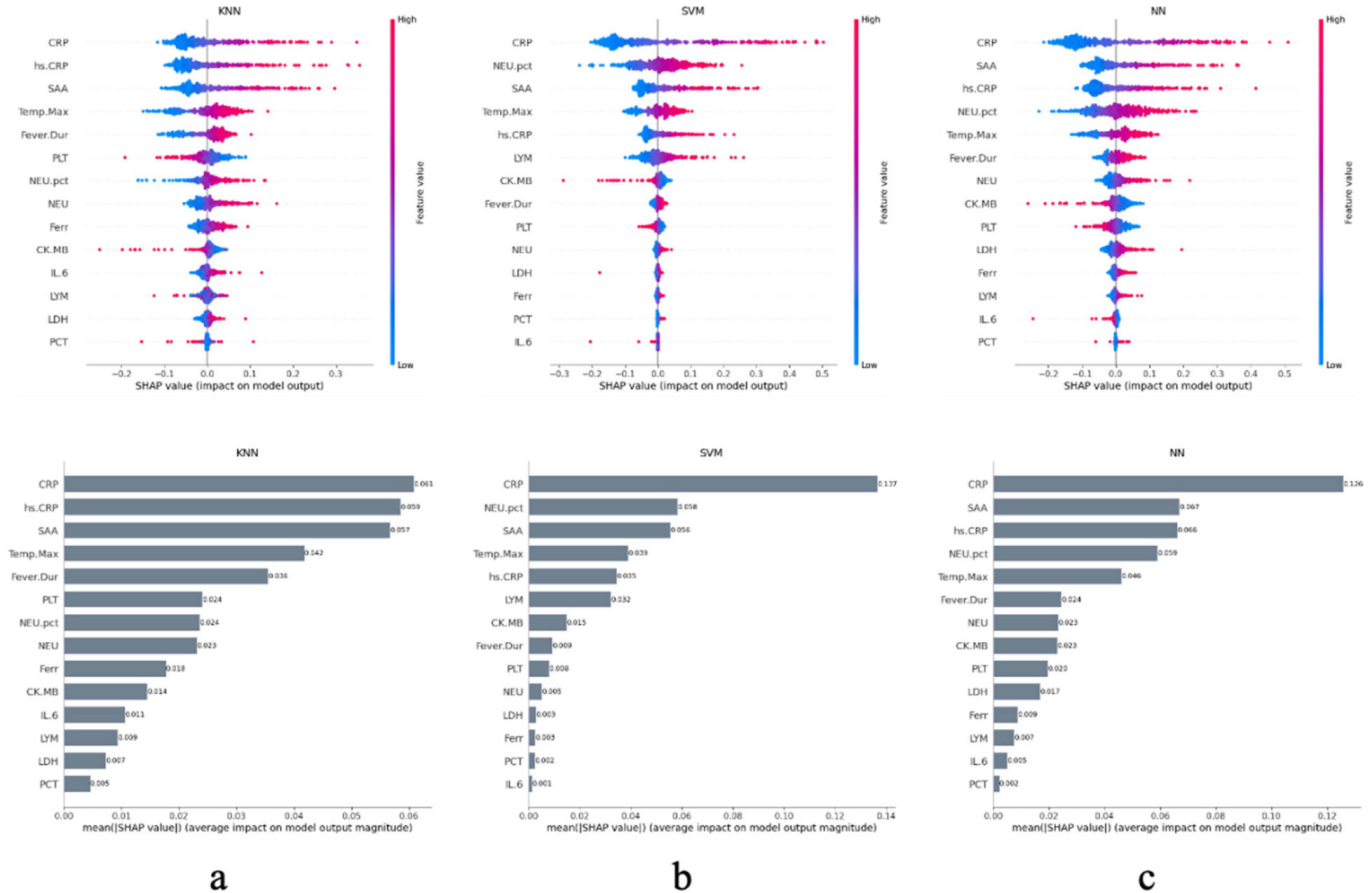
SHAP plot showing the impact of clinical features on the diagnosis of MPP in the **(a)** KNN, **(b)** SVM and **(c)** NN.

Based on these results, SHAP analysis was further employed to identify variables with critical discriminative power in each model. Initially, all 14 variables were included to construct baseline models for each algorithm, and SHAP values were calculated for every sample. The mean absolute SHAP values were then used to measure feature importance. The results demonstrated that SAA, CRP, hs.CRP, CK-MB, Temp.Max, ferritin, and NEU.pct consistently ranked among the top features across multiple models, highlighting their central role in prediction (Figure 1).

Furthermore, forward stepwise feature selection was performed according to SHAP rankings to construct the optimal feature subset (see Methods). At each step, a new variable was added, and the AUC on the training set was evaluated using five-fold cross-validation, with the AUC trend plotted against the number of variables. Ultimately, different models selected distinct subsets of features, suggesting variability in feature utilization and discriminative pathways across algorithms (Table 2).

**Table 2:** Characteristics selection of different models.

Model	Characteristics
KNN	“Temp.Max”, “SAA”, “CRP”, “hs.CRP”
SVM	“NEU.pct”, “SAA”, “CRP”, “Temp.Max”, “hs.CRP”
NN	“NEU.pct”, “SAA”, “CRP”, “hs.CRP”, “Temp.Max”, “Fever.Dur”, “NEU”, “CK.MB”

Predictive performance of ML models.

**Figure 2:**
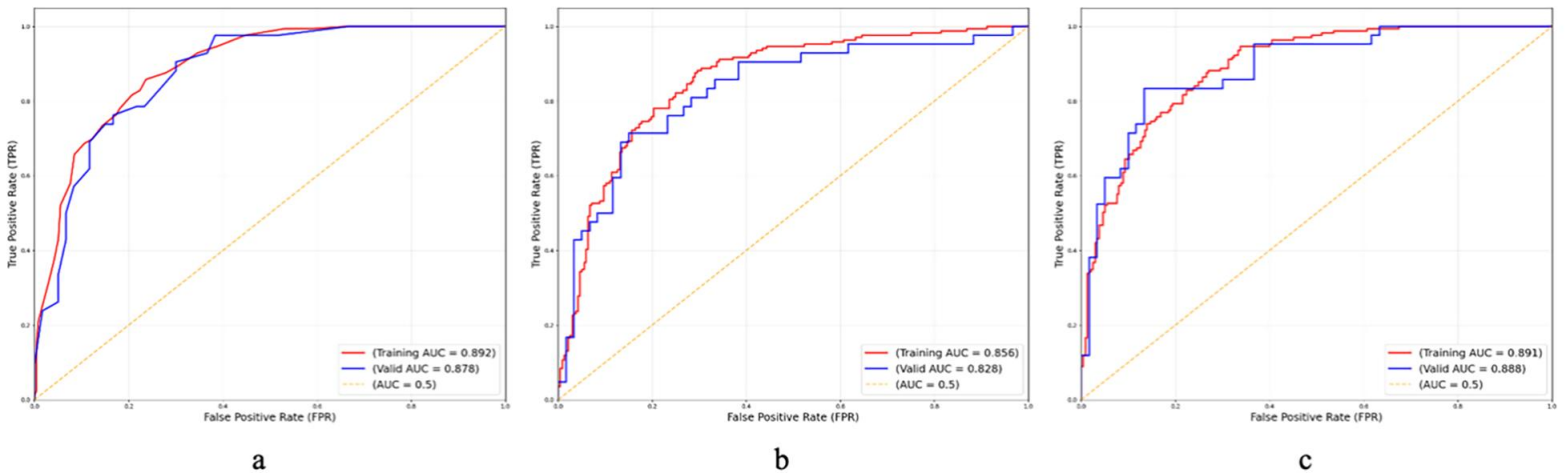
The ROC of the three ML models. The plots show the ROC of **(a)** KNN, **(b)** SVM, and **(c)** NN. These curves depict the performance of the models on both the training and validation set.

In this study, we compared the performance of multiple ML algorithms for predicting the risk of atelectasis in children with MPP. The classification performance of different models in the training and validation sets is presented in Tables 3, 4, with the corresponding ROC curves shown in Figure 2.

**Table 3:** Performance of different models on the training set.

Model	Accuracy	Precision	Recall	F1 score	AUC (ROC)	Specificity	PPV	NPV
KNN	0.81 (0.78, 0.83)	0.79 (0.74, 0.82)	0.73 (0.68, 0.79)	0.76 (0.72, 0.79)	0.89 (0.88, 0.91)	0.86 (0.83, 0.89)	0.79 (0.74, 0.82)	0.82 (0.79, 0.85)
SVM	0.78 (0.76, 0.80)	0.73 (0.70, 0.75)	0.75 (0.72, 0.79)	0.74 (0.71, 0.77)	0.86 (0.84, 0.88)	0.80 (0.78, 0.82)	0.73 (0.70, 0.75)	0.82 (0.80, 0.84)
NN	0.81 (0.75, 0.84)	0.79 (0.72, 0.85)	0.72 (0.65, 0.78)	0.76 (0.68, 0.80)	0.89 (0.86, 0.92)	0.87 (0.82, 0.90)	0.79 (0.72, 0.85)	0.81 (0.77, 0.85)

**Table 4:** Performance of different models on the validation set.

Model	Accuracy	Precision	Recall	F1 score	AUC (ROC)	Specificity	PPV	NPV
KNN	0.80 (0.72, 0.87)	0.76 (0.62, 0.89)	0.74 (0.60, 0.87)	0.75 (0.63, 0.84)	0.88 (0.81, 0.94)	0.83 (0.73, 0.93)	0.76 (0.62, 0.89)	0.82 (0.72, 0.91)
SVM	0.78 (0.70, 0.85)	0.78 (0.62, 0.91)	0.65 (0.50, 0.79)	0.70 (0.58, 0.81)	0.83 (0.75, 0.91)	0.87 (0.78, 0.95)	0.78 (0.62, 0.91)	0.78 (0.68, 0.87)
NN	0.82 (0.75, 0.89)	0.82 (0.69, 0.93)	0.74 (0.60, 0.88)	0.78 (0.66, 0.87)	0.89 (0.82, 0.95)	0.88 (0.80, 0.96)	0.82 (0.69, 0.93)	0.83 (0.73, 0.92)

Table 3 summarizes the classification performance of three ML models (KNN, SVM, and NN) on the training set. Overall, both KNN and NN achieved the best performance, with an accuracy of 0.81, an F1 score of 0.76, and an AUC of 0.89, indicating strong discriminative ability and good fitting performance. The SVM model performed slightly lower, achieving an AUC of 0.86 and an accuracy of 0.78, but still demonstrated reasonable stability. The specificity and NPV of all models were above 0.80, suggesting high reliability in identifying negative cases.

Performance on the validation set (Table 4) further demonstrated the models' generalization capabilities. The NN model again outperformed the others, with an accuracy of 0.82, an F1 score of 0.78, and an AUC of 0.89, showing excellent robustness and discriminative power on unseen data. The KNN model followed closely, with an accuracy of 0.80 and an AUC of 0.88, indicating good generalization. Although SVM achieved a slightly lower accuracy (0.78) and AUC (0.83), it maintained the highest specificity (0.87), suggesting superior performance in distinguishing negative samples.

Figure 2 illustrates the ROC curves of the models on both the training and validation sets. All three models demonstrated ROC curves well above the diagonal line of random guessing, with the NN curve closest to the top-left corner, indicating optimal overall performance. The minimal difference between the training and validation AUCs suggests no significant overfitting and good generalization capability.

In summary, all three models showed stable and accurate performance across both datasets. The NN model exhibited the best overall performance, followed by KNN, while SVM demonstrated strong specificity. These findings indicate that ML models are effective for disease risk prediction and hold promising potential for clinical application.

## Discussion

This study systematically compared the performance of three classical ML models in disease risk prediction. The results demonstrated that all models achieved high discriminative power on both training and validation sets, with the NN model performing best overall, followed by KNN, while SVM exhibited an advantage in specificity.

The superior performance of the NN model can be attributed to its strong nonlinear feature mapping capability, enabling it to capture complex interactions among multidimensional clinical variables (28). Moreover, the minimal difference in AUC between training and validation sets indicates robust generalization ability. Although KNN showed comparable performance, it may be affected by neighborhood selection and data distribution, leading to slightly inferior results in complex datasets. The SVM model, despite slightly lower overall accuracy, achieved the highest specificity, suggesting its potential utility in identifying low-risk or negative cases.

Overall, the findings highlight the feasibility and clinical applicability of ML for disease risk prediction. Future studies should explore the integration of ensemble and deep learning methods to further enhance feature representation and validate model generalizability across multicenter and prospective datasets.

## Conclusion

This study systematically evaluated the performance of three ML models—KNN, SVM, and NN—in predicting the risk of MPP-associated atelectasis. The results indicate that while all models demonstrated good discriminative ability, the neural network (NN) model was superior in overall performance (AUC and accuracy). This is likely attributable to the NN model's inherent multi-layer non-linear transformation capabilities, which enable it to better capture the complex, high-dimensional interactions among clinical features throughout the course of MPP.

Concurrently, the optimal feature subsets relied upon by different models varied. For instance, both NN and SVM assigned high importance to neutrophil percentage (NEU.pct), whereas the KNN model depended more on body temperature and inflammatory markers. This reveals that different algorithms may employ distinct “cognitive” pathways for capturing disease signals. Despite these differences, SHAP analysis uncovered a cross-model consensus: inflammation-related indicators such as SAA, CRP, and NEU.pct are the cornerstones for predicting atelectasis. This finding significantly enhances the credibility of these biomarkers for clinical decision-making.

Notably, while the SVM model had a slightly lower overall AUC, it achieved the highest specificity. This implies that SVM is more reliable for “ruling out” patients without atelectasis, offering unique value in clinical scenarios where minimizing false positives—and thus avoiding over-intervention in low-risk patients—is paramount.

The primary limitation of this study is its single-center, retrospective design. Future work should validate these findings in larger, multicenter, prospective cohorts and explore ensemble strategies that combine the strengths of different model types. In conclusion, our research confirms that the choice of a ML model can be optimized based on specific clinical needs: a neural network is preferred for the highest overall predictive accuracy, while the SVM model presents a distinct advantage when high specificity is required. This diversified, model-based selection strategy, combined with the transparent explanations provided by SHAP, powerfully facilitates the translation and application of ML models into the clinical practice of pediatric respiratory diseases.

## Data Availability

The raw data supporting the conclusions of this article will be made available by the authors, without undue reservation.
